# Comparison of Live Versus Online Instruction of a Novel Soft Skills Course in Mongolia

**DOI:** 10.7759/cureus.1900

**Published:** 2017-11-30

**Authors:** Aditya Mahadevan, Matthew C Strehlow, Khandregzen Dorjsuren, Jennifer A Newberry

**Affiliations:** 1 Department of Biology, University California San Diego; 2 Department of Emergency Medicine, Stanford University School of Medicine; 3 School of Medicine, Mongolian National University

**Keywords:** mongolia, education, online, classroom, soft skills, international, comfort, knowledge, mindset, satisfaction

## Abstract

Background

Soft skills are essential for employee success in the global marketplace; however, many developing countries lack content experts to provide the requisite instruction to an emerging workforce. One possible solution is to use an online, open-access curriculum. To date, no studies on soft skills curricula using an online learning platform have been undertaken in Mongolia.

Objective

To evaluate the efficacy of an online versus classroom platform to deliver a novel soft skills course in Mongolia.

Methods

A series of eight lectures along with corresponding surveys and multiple choice question tests were developed and translated into the Mongolian language. Two different delivery modalities, online and traditional classroom lectures, were then compared for knowledge gain, comfort level, and satisfaction. Knowledge gain and comfort level were assessed pre- and post-course, while satisfaction was assessed only post-course.

Results

Enrollment in the online and classroom courses was 89 students and 291 students, respectively. Sixty-two online students (68% female) and 114 classroom students (77% female) completed the entire course and took the post-test. The online cohort had higher pre-test scores than the classroom cohort (46.4% and 37.3%, respectively, p < 0.01). The online cohort’s overall knowledge gain was not significant (0.4%, p=0.87), but the classroom cohort’s knowledge gain was significant (13.9%, p < 0.01). Both the online and classroom cohorts demonstrated significant improvement in overall comfort level for all soft skills topics (p < 0.01). Both cohorts were also highly satisfied with the course, as assessed on a Likert scale (4.59 for online, 4.40 for classroom).

Conclusion

The study compared two cohorts of Mongolian college students who took either an online or classroom-based soft skills course, and it was found that knowledge gain was significantly higher for the classroom group, while comfort and satisfaction with individual course topics was comparable.

## Introduction

It is well recognized by global and US-based companies that soft skills are essential for employee success [1]. Prior research indicates that 85% of job success comes from well-developed people skills (‘soft skills’) while only 15% originates from knowledge and technical skills (‘hard skills’) [2]. Yet, in many developing countries, soft skills education is often neglected in favor of traditional subjects [3].

One barrier to teaching soft skills in developing countries is a lack of content experts to provide the requisite instruction. Simply sending educators abroad may not be effective as they must overcome language and cultural barriers, and their curriculum must be adapted to the local context. In addition, the resources required for foreign educators to travel and teach international courses may render such efforts unsustainable.

One possible solution is to employ an online, open-access curriculum. The benefits of online education include flexibility, convenience, accessibility, interactivity, and adaptability to different learning styles [4]. Consequently, the growth of online education has been outpacing traditional education since 2002 [5].

Research in online education has focused almost exclusively on knowledge gain. A recent meta-analysis, reviewing 126 studies of online learning interventions, demonstrated that all but two were associated with knowledge improvement [6]. However, there is limited online education research evaluating students’ comfort levels and confidence with course content [7-8]. Since confidence with material is moderately correlated with problem-solving and memory, it is important to measure this parameter when assessing online courses [9]. A lack of comfort with course material may lead to difficulty applying new knowledge.

Since most online education research has been conducted in developed countries, another important consideration is its generalizability to the developing world. To date, no studies on soft skills curricula using an online learning platform have been undertaken in Mongolia [6].

This study aims to evaluate the efficacy of deploying a novel soft skills and social psychology course in Mongolia. We compared two different modalities, online versus traditional classroom lectures for knowledge gain, comfort level, and satisfaction. If an online soft skills curriculum proves effective, then such courses can be scaled in developing countries to meet the critical need for soft skills education.

## Materials and methods

Setting

**Figure 1:**
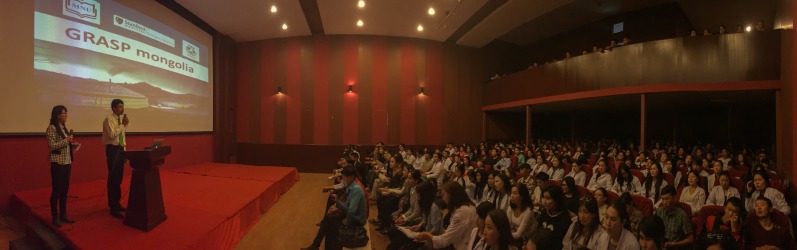
In-person Classroom Course in Mongolia

The course was taught to college students from Mongolian National University in Ulaan Bataar, Mongolia, over one week in September 2015. Mongolia National University is the largest private university in Mongolia, with over 10,000 students and 80 undergraduate and graduate programs (Figure 1).

Curriculum development

We developed a series of eight lectures covering key topics on soft skills and wellness including mindset, teamwork, interviews, networking, self-belief, productivity, sleep, and physical wellness. All topics, except for sleep and physical wellness, were chosen based on an international employer questionnaire encompassing competencies expected of graduates, including key transferrable soft skills and competencies for employability [10].

For both the online and traditional classroom courses, the same lecture slides were used. To create the online course, each topic’s lecture slides were sequentially video recorded with an accompanying English narration. Subsequently, a native Mongolian speaker provided consecutive translation of the English narration into the Mongolian language. The video lectures and accompanying Mongolian translation were combined using screen capture software (Camtasia®, TechSmith Corporation, USA). The lectures were uploaded to an online classroom platform (Lensoo®, Lensoo, Inc. USA) that students accessed at their own pace (http://bit.ly/2ry6hd5). The traditional classroom-based course was taught in English with contemporaneous translation by a Mongolian interpreter. The eight lectures comprising the classroom course were given over two hours.

Study design

Students were given the option to take the course either online or in the traditional classroom setting, based on both their personal interest and their access to the internet (either private or on campus). The students in the online group had access to the internet-based course for one week.

To test the students’ knowledge gain, both cohorts were given a multiple-choice question (MCQ) pre-test and post-test. The MCQs were derived directly from the lecture material. Ten college students field-tested the MCQs in the United States beforehand to establish readability and face validity.

The students were given a survey before and after the course to determine their comfort level with individual course topics. Satisfaction with the course was also assessed with the post-course survey. The surveys used a Likert scale with 1 being the least comfortable or satisfied and 5 being the most.

Both the survey and the MCQs were translated into the Mongolian language by a professor at Mongolia National University and reviewed by three other professors to ensure that the translated questions remained true to their original intent.

Data analysis

Paired t-tests were used to compare pre-test and post-test scores. Independent t-tests were used to compare knowledge gain between the online and traditional classroom groups. We considered a p-value < 0.05 to be statistically significant. All analysis was completed using STATA 13.1 (StataCorp LLC, USA).

## Results

Baseline characteristics

Enrollment in the online and classroom courses was 89 students and 291 students, respectively. The median age of all participants was 19 (IQR 18-20) and 74% were female. Sixty-two online students (68% female) and 114 classroom students (77% female) completed the entire course and took the post-test. Attrition rates were 30% (N=27) for the online group and 61% (N=177) for the classroom group.

Knowledge gain

The online cohort had higher pre-test scores than the classroom cohort (46.4% and 37.3%, respectively, p < 0.01). The online cohort’s overall test-score improvement was not significant (0.4%, p = 0.87) in contrast to the classroom cohort’s test-score improvement (13.9%, p < 0.01) (Table 1).

**Table 1:** Comparison of Knowledge Gain by Delivery Method

	Pre-test		Post-test		Knowledge		p-value
Online (N=62)	46.4%	42.3%-50.5%	46.8%	42.9%-50.7%	0.4%	-4.6%-5.5%	.87
Classroom (N=114)	37.3%	34.2%-40.4%	51.2%	47.8%-54.5%	13.9%	10.3%-17.5%	<.01

Comfort level

All online participants completed the surveys on their comfort with the materials. The classroom group only had 82 participants who completed both the pre and post-course survey. Both the online and classroom cohorts demonstrated significant improvement in overall comfort level for all of the soft-skills topics (p < 0.01). The individual topic of sleep was associated with the greatest overall increase in comfort level for both groups (Table 2).

**Table 2:** Online and Classroom Comfort Level *Likert scale where 1 = least comfortable to 5 = most comfortable. **p< .01

Topic	Pre-Course ^*^		Post-Course^*^		Change	
	Online	Classroom	Online	Classroom	Online	Classroom
Mindset	4.31	4.11	4.58	4.44	.27	.33
Teamwork	4.19	4.23	4.50	4.31	.31	.08
Confidence	4.08	4.16	4.52	4.39	.44	.23
Interviews	4.11	4.12	4.39	4.46	.28	.34
Networking	3.69	3.56	4.16	3.96	.47	.40
Productivity	4.26	4.28	4.48	4.35	.22	.07
Sleep	3.61	3.64	4.35	4.26	.74	.62
Physical well being	3.69	3.97	4.15	4.14	.46	.17
Average	4.00	4.01	4.39	4.30	0.39^**^	0.29^**^

Satisfaction

Overall, the students were highly satisfied with the course delivered by both the online and classroom methods. The subjects in the online group were more likely to recommend the course (Table 3).

**Table 3:** Comparison of Satisfaction Surveys from Online and Classroom groups *Likert scale where 1 = strongly disagree to 5 = strongly agree.

Question	Online (N=62)*	Classroom (N=113)*
Overall satisfaction	4.70	4.66
Clarity	4.61	4.45
Pacing	4.34	3.97
Relevancy	4.76	4.76
Likelihood to recommend	4.53	4.14

## Discussion

The study evaluated the efficacy of an online social psychology and soft skills course compared with an identical classroom-based course in Ulaan Bataar, Mongolia. This was the first such study conducted at a college campus in Mongolia, where only 21.4% of the population had access to the internet in 2015 [11]. Relative gains in knowledge, comfort, and differences in satisfaction were analyzed.

The classroom group demonstrated a significant gain in overall knowledge, in spite of participating in the first such soft-skills course ever taught at their college campus. However, the online group failed to demonstrate a statistically significant improvement in overall knowledge, suggesting diminished efficacy compared to the classroom-based course. Further, the findings from our pilot study do not lend further support to prior research which demonstrated that soft skills courses, like ours, are conducive to online delivery [12].

Both groups showed significant improvement in their overall comfort levels with the soft skills-focused topics. At the conclusion of the course, the online group generally rated their comfort level as higher than the classroom group for most of the individual course topics. However, this did not translate into superior knowledge gains in the areas where they stated that they felt more comfortable.

Both groups described high satisfaction with the overall course and individual measures including relevancy, clarity, and pacing. This is in contrast to a previous study which found that online courses are associated with significantly lower satisfaction than their classroom counterparts [13].

This study has a number of limitations. First, many students did not complete the entirety of the course and assessments, especially in the classroom cohort. This was mainly due to an unanticipated schedule overlap with students’ core course requirements. There was also not enough data on personality engagement and demographics to do a sensitivity analysis, which could have yielded valuable information regarding the population that did not finish the course. Secondly, students were not randomly assigned to their learning groups leading to potential selection bias and a likely overestimation of satisfaction with the online course as students “opted in” to this modality. This may have contributed to the online cohort having higher baseline knowledge scores. Thirdly, there was no verification that online students attentively watched the videos or that they took the post-test immediately after completion of the course.

## Conclusions

Our study sought to determine the efficacy of an online soft skills course in comparison to an identical classroom-based course in an international setting. We determined that knowledge gains were significantly higher for the classroom group, while comfort and satisfaction with individual course topics was comparable. The vast majority of participants in both groups found the course’s subject matter relevant. While approximately 25% of US students have taken a course online, internet-based education has yet to take hold in Mongolia. Future studies should focus on how to improve the efficacy of online curricula in this country, as an open-access educational approach has the potential to assist far more participants in this rural nation.

## References

[1] Cunningham W, Villaseñor P (2014). Employer demands, and implications for public skills development policy. Policy Research Working Papers.

[2] Wei LS, Chea CC, Aziz RC (2017). National Soft Skills Association: where did this come from?. PCF8.

[3] (2017). 2016-2017 Talent Shortage Survey. http://manpowergroup.com/talent-shortage-2016.

[4] Aggarwal R, Gupte N, Kass N (2011). A comparison of online versus on-site training in health research methodology: a randomized study. BMC Med Educ.

[5] Allen E, Seaman J (2017). Going the distance: online education in the United States, 2011. Baboon Survey Research Group.

[6] Cook DA, Levinson AJ, Garside S, Dupras DM, Erwin PJ, Montori VM (2008). Internet-based learning in the health professions: a meta-analysis. JAMA.

[7] McLaren CH (2004). A comparison of student persistence and performance in online and classroom business statistics experiences. Decis Sci J Innov Educ.

[8] Merisotis JP, Phipps RA (2017). What’s the difference?: a review of contemporary research on the effectiveness of distance learning in higher education. IHEP.

[9] Stankov L, Lee J (2017). Confidence and cognitive test performance. J Educ Psychol.

[10] Andrews J, Higson H (2008). Graduate employability, ‘soft skills’ versus ‘hard’ business knowledge: a European study. Higher Educ.

[11] Mongolia Mongolia (2017). The global economy - Mongolia internet users. http://www.theglobaleconomy.com/Mongolia/Internet_users/. Accessed.

[12] Callister RR, Love MS (2016). A comparison of learning outcomes in skills-based courses: online versus face-to-face formats. Decis Sci J Innov Educ.

[13] Summers JJ, Waigandt A, Whittaker TA (2005). Comparison of student achievement and satisfaction in an online versus a traditional face-to-face statistics class. Innov High Educ.

